# Transparent non-cubic laser ceramics with fine microstructure

**DOI:** 10.1038/s41598-019-46616-8

**Published:** 2019-07-16

**Authors:** Hiroaki Furuse, Naohiro Horiuchi, Byung-Nam Kim

**Affiliations:** 1 Kitami Institute of Technology, 165 Koen-cho, Kitami, Hokkaido 090-8507 Japan; 2 Tokyo Medical and Dental University, 2-3-10 Kanda-Surugadai, Chiyoda-ku, Tokyo, 101-0062 Japan; 3 National Institute for Materials Science, 1-2-1 Sengen, Tsukuba, Ibaraki 305-047 Japan

**Keywords:** Solid-state lasers, Solid-state lasers

## Abstract

Transparent polycrystalline ceramics with cubic crystal structure have played important roles in a wide variety of solid-state laser applications, whereas for non-cubic structures, single crystal only has been used. For further progress in optical technologies, effective materials beyond the current limitations are necessary. Here we report a new type of non-cubic ceramic laser material that overturns conventional common sense. It is hexagonal Nd-doped fluorapatite (Nd:FAP) ceramics with an optical quality comparable to single crystal while having random crystal orientation. It is composed of ultrafine grains with a loss coefficient of 0.18 cm^−1^ at a lasing wavelength of 1063 nm, and its laser oscillation was demonstrated. This is the first verification of lasing in randomly oriented non-cubic ceramics. Laser oscillation in the non-cubic ceramics was realized through both advanced liquid-phase nano-powder synthesis technology and highly controlled pulsed-current sintering techniques. Our findings should open new avenues for future solid-state laser and optical applications.

## Introduction

Transparent ceramics with high optical quality are used in various applications, including industrial processing, medicine, aerospace, and high-power laser physics for inertial laser fusion owing to the superior features of ceramics such as enlargement, ease of compositing, uniformity, and high concentration of soluble active elements^1^. Most transparent ceramics have a cubic crystal structure, and development of novel ceramics with advanced mid-infrared lasing, magneto-optic properties, scintillation capability or illumination is still being actively pursued.

Here we report on a new type of laser ceramic material: Nd-doped fluorapatite (Nd:FAP) ceramics with a hexagonal crystal structure. The microstructure has a random crystal orientation but little birefringent scattering due to sufficiently small grain sizes. The average grain size is only 140 nm, and the in-line transmittance for a 1-mm thick sample reaches 87.4% at 1063-nm laser wavelength, corresponding to 98.7% of the theoretical transmittance. FAP is a promising candidate as an effective laser host material for an inertial laser fusion driver^2^, and is also a potential biomaterial for osteogenic bone. We believe that the novel non-cubic laser ceramics will lead to significant future development in optical and medical fields.

In general, it is not easy to achieve laser-grade transparency in sintered polycrystalline ceramics. After sintering, various defects remain, such as pores, vacancies, secondary phases, impurities, grain boundaries and surface roughness which act as a light scattering source^3^. These sintering defects have been greatly reduced to the limit by advanced control technologies for both powder synthesis and ceramic sintering, to yield high-quality transparent laser ceramics^4,5^. In fact, such high power as 100-kW output (continuous wave, CW)^6^, and 100-J output pulse energy of a ns pulse^7^, have been achieved for Nd:YAG and Yb:YAG ceramics, respectively. Conventionally, laser ceramics are sintered at high temperatures, so that the final grain size for most cases is >2 μm, which is equal to or greater than typical laser wavelengths. For this reason, conventional laser ceramics are limited to cubic crystals, since non-cubic grains of a comparable size induce light scattering, called Mie scattering.

The scattering in non-cubic ceramics can be decreased by reducing the mismatch of crystal orientation between grains. Suzuki *et al*. developed a method of controlling crystal orientation using a strong magnetic field^8^. Building on this accomplishment, Akiyama *et al*. developed non-cubic Nd:FAP laser ceramics with a slope efficiency of 2.6% upon laser oscillation^9^. The Nd:FAP ceramics sintered at high temperatures (1600 °C) under ambient pressure exhibited an average loss coefficient of 1.5 cm^−1^. Their development is the first achievement of non-cubic laser ceramics.

**Figure 1:**
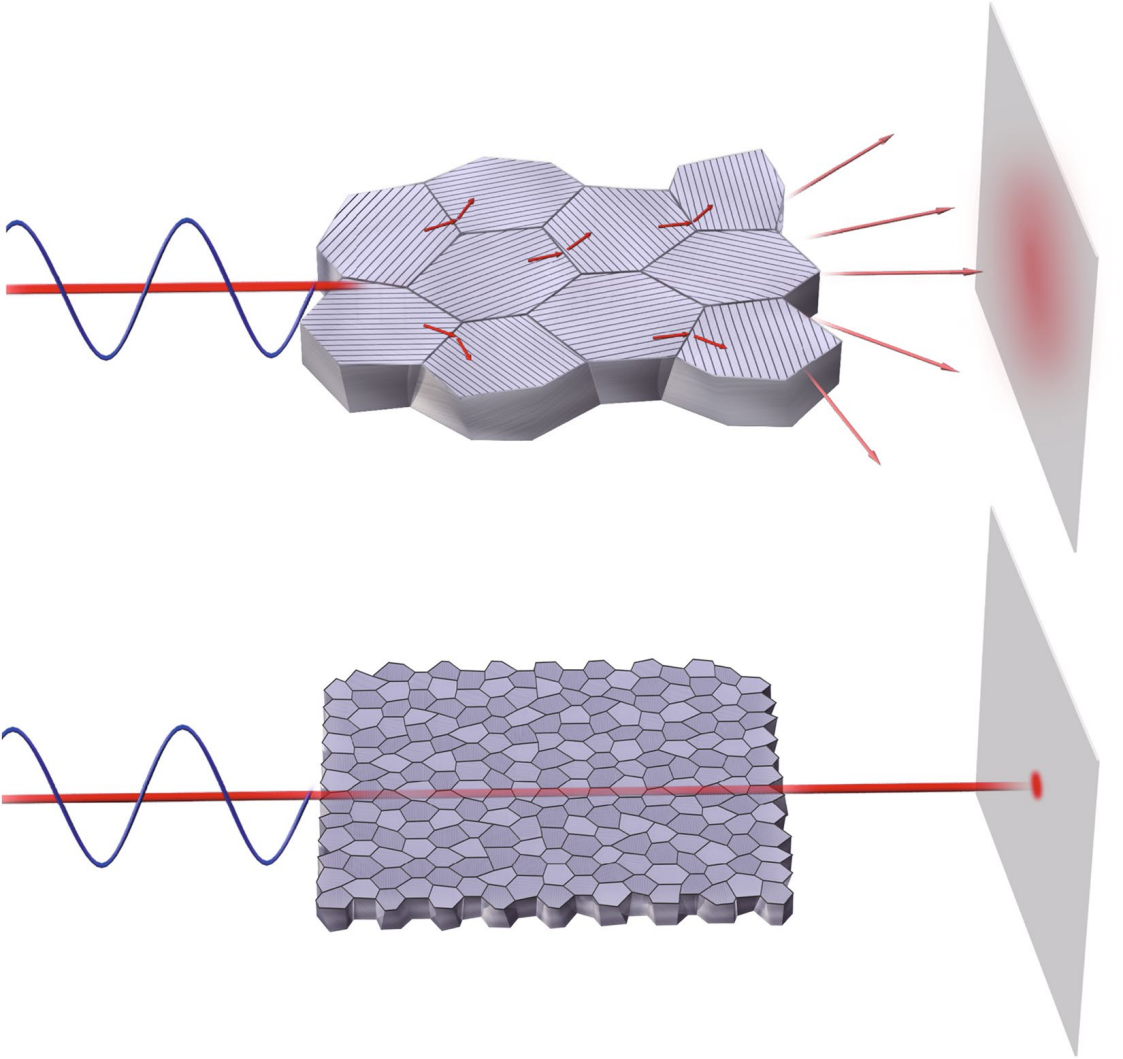
Schematics of optical scattering in fine-grained non-cubic ceramics. When the grain size is comparable to the wavelength of light, Mie scattering occurs at grain boundaries due to a discrepancy between refractive indices of different crystal orientations. However, when the grain size is sufficiently small compared to the wavelength, Mie scattering at grain boundaries is suppressed to permit light passing through the sample.

Another approach for reducing the light scattering in non-cubic ceramics is to make the microstructure fine and dense. When the non-cubic grains are sufficiently small compared to the wavelength of light, Mie scattering at grain boundaries disappears; consequently the transmittance is enhanced regardless of the crystal orientation of the grains (Fig. 1). The pores remaining after sintering also significantly deteriorate the transmittance. A pore volume of only 0.5% can make a sample completely opaque. Hence, for randomly oriented transparent non-cubic ceramics, compatibility with fine microstructure and high density is required.

Kim *et al*. fabricated transparent non-cubic alumina ceramics with an average grain size of 230 nm by using spark plasma sintering (SPS)^10^. SPS is a sintering technique where powder is heated directly by a pulsed electric current under uniaxial pressing. As compared to conventional techniques such as pressureless sintering, hot pressing and hot isostatic pressing, SPS has a unique advantage for synthesizing fine-grained transparent ceramics. The external field of electric current and pressure enables rapid heating, short sintering times and densification at low temperatures^11^. These characteristics can suppress grain growth and enhance densification during sintering to yield a fine-grained and highly dense microstructure that reduces light scattering from anisotropic grains and remaining pores. Penilla *et al*. also fabricated Nd-doped transparent alumina by using SPS and observed optical amplification^12^.

Besides alumina, Kim *et al*. obtained highly transparent hydroxyapatite ceramics using a SiC mould during SPS^13^. The microstructure had a grain size of 120 nm, and the transmittance was close to a theoretical value. Wu *et al*. reported Yb-doped apatite ceramics with a transmittance of about 80% at 1-μm wavelength and confirmed a fluorescence^14,15^. To our knowledge, however, laser oscillation has not yet been demonstrated for randomly oriented non-cubic ceramics. To demonstrate laser oscillation for such randomly oriented ceramics, both fine grains and high density should be attained simultaneously.

The main purpose of this study is to demonstrate laser oscillation from randomly oriented non-cubic polycrystalline ceramics for the first time. In this work, we synthesized Nd:FAP nano-powder via a liquid-phase synthesis to realize transparent non-cubic ceramics. Highly densified transparent Nd:FAP ceramics with a fine microstructure were sintered by using an SPS technique. For the Nd:FAP powder and ceramics, we investigated the microstructure, also acquiring the optical transmittance, crystal structures, fluorescent properties and laser performance.

## Results and Discussion

### Microstructural and optical characteristics

**Figure 2:**
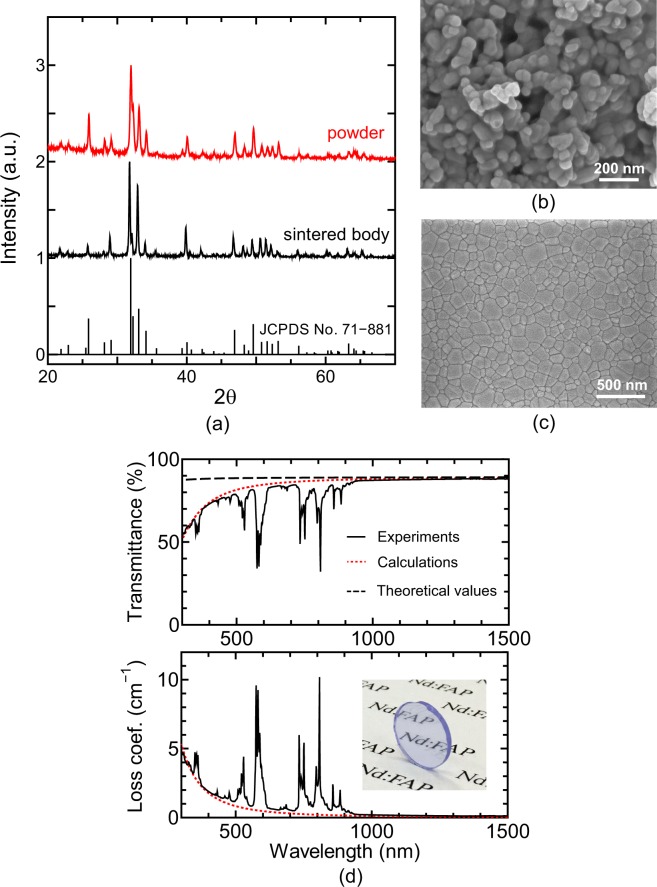
Microstructural and transmittance characteristics of fine-grained Nd:FAP ceramics. (**a**) X-ray diffraction pattern of synthesized Nd-doped FAP powder and sintered Nd:FAP ceramics, (**b**) FE-SEM image of Nd:FAP powder, (**c**) FE-SEM image of the polished Nd:FAP ceramics, and (**d**) transmitted spectrum and loss coefficient of Nd:FAP ceramics. The dashed line is the theoretical transmittance, and the red dotted lines are calculated ones. The inset shows a 1-mm thick Nd:FAP ceramic sample after polishing.

Figure 2a shows the X-ray diffraction spectra for the Nd:FAP powder and sintered body. The diffraction angles and relative peak strengths for the Nd:FAP ceramics coincide well with those of the standard FAP powder (JCPDS cards No. 71-881), indicating that the Nd:FAP ceramics are single phase and consist of randomly oriented grains. The relatively lower and higher peak strengths at 26° and 40°, respectively, may indicate slightly oriented microstructures. During SPS, the application of uniaxial pressure can make the microstructure slightly oriented perpendicular to the pressing direction^16^. Note that the X-ray diffraction spectra for oriented Nd:FAP (aligned using a magnetic field) shows strong diffraction peaks in a specific plane direction^17^. Figure 2b,c display the microstructure of the synthesized powder and sintered body. The powder has a primary particle size of about 50 nm and a nearly spherical morphology. For the sintered ceramics, the microstructure is almost fully dense and no secondary phases are observed. The average grain size is 140 nm, which is about one order of magnitude smaller than the lasing wavelength (1063 nm). Thus, the present powder processing and sintering techniques are highly effective in fabricating fine-grained transparent Nd:FAP ceramics.

Figure 2d shows the in-line transmitted spectra and total loss coefficient spectra *δ*(*λ*) for the Nd:FAP ceramics shown in the inset. *δ*(*λ*) is a sum of the absorption coefficient *α* and scattering coefficient *γ*. The black dashed line represents the theoretical transmittance of FAP, calculated from the average refractive index dispersion, *n*_av_(*λ*) (see Supplementary Fig. S1). The scattering coefficient *γ*(*λ*) was estimated by fitting the experimental loss coefficient curve, excluding absorption peaks under an assumption of *α* = 0, and the optical transmittance was calculated from equation (3) in Section Methods. Both the fitted *γ*(*λ*) and calculated transmittance are represented as dotted red lines in Fig. 2d.

In transparent non-cubic ceramics, two scattering sources can be considered: birefringent grains and pores. Since hydroxyapatite ceramics prepared in a way similar to the present techniques exhibited nano-sized pores of 5–30 nm^13^, it is likely that the Nd:FAP ceramics also contain such pores. The nano-sized pores are considerably smaller than the wavelengths to cause Rayleigh scattering *γ*_p_^18^, whereas the birefringent grains comparable to the wavelengths cause Mie scattering *γ*_g_^19^. The two scattering coefficients can be represented theoretically as,

1 $${\gamma }_{{\rm{g}}}(\lambda )=\frac{3{\pi }^{2}{d}_{{\rm{g}}}{\rm{\Delta }}{n}_{{\rm{g}}}^{2}}{{\lambda }^{2}}{V}_{g},$$

2 $${\gamma }_{{\rm{p}}}(\lambda )=\frac{16{\pi }^{4}{d}_{{\rm{p}}}^{3}{\rm{\Delta }}{n}_{p}^{2}{n}_{{\rm{av}}}^{2}}{9{\lambda }^{4}}{V}_{{\rm{p}}},$$

where *d*_g_ and *d*_p_ are the grain and pore sizes, Δ*n*_g_ is an average difference in the refractive indices of birefringent FAP grains, Δ*n*_p_ (=*n*_av_ − 1) is a refractive-index difference between a pore (=1) and the grains (*n*_av_) (see Supplementary Table 1), *V*_g_ and *V*_p_ are the effective volume fraction of the grains and pores, respectively. Since the measured scattering is as a sum of the two Mie and Rayleigh scatterings (*γ* = *γ*_g_ + *γ*_p_), equations (1) and (2) were used to fit the experimental *γ*. For the fitting, the size and the volume density of the grains were set to *d*_g_ = 140 nm and *V*_g_ = 0.5, respectively. The fitting is in good agreement with the experimental *γ* for *d*_p_ = 24 nm and *V*_p_ = 0.001. This indicates that the nano-sized pores may remain in the grain boundary triple points or within the grain at a density of about 0.1%. As a value of *V*_g_, Apetz and van Bruggen^19^ assumed 0.5 for fully dense non-cubic ceramics, and Kim *et al*.^20^ obtained experimentally 0.3–0.4 for alumina. For the present FAP, the *V*_g_-value in the scattering model of Apetz and van Bruggen was employed.

From the experimental results, the total loss coefficient at the laser wavelength *λ* = 1063 nm was *δ* = 0.18 cm^−1^. The in-line transmittance at the laser wavelength was 87%, which corresponds to 98% of the theoretical transmittance. Note that the scattering coefficient decreases upon shifting to longer wavelengths by suppressing Mie scattering. In particular, the measured scattering coefficient at *λ* = 1500 nm was reduced to 0.13 cm^−1^, and the in-line transmittance increased to 99.1% with respect to the theoretical transmittance. This fact suggests that the fine-grained FAP ceramics may be a suitable new material for mid-infrared optics.

### Spectroscopic and laser properties

**Figure 3:**
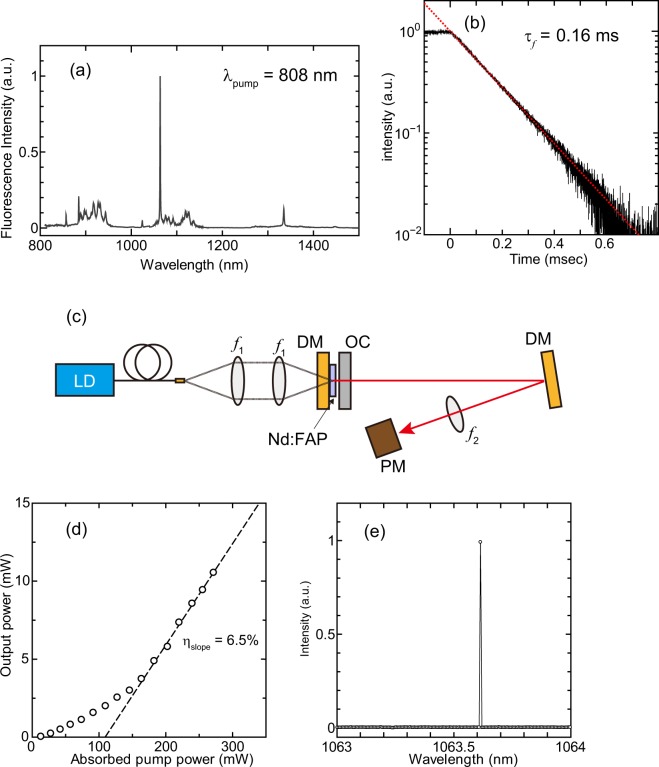
Spectroscopic and laser properties of Nd:FAP ceramics. (**a**) Fluorescence spectra pumped by 807 nm laser diode, (**b**) Fluorescence decay curve and calculated lifetime, (**c**) Schematic diagram of experimental setup for laser oscillation, (**d**) Laser output power as a function of absorbed pump power at 1 ms-pulse duration and 10 Hz-repetition rate, and (**e**) Typical lasing spectrum of Nd:FAP laser oscillation for single pulse at *P*_pump_ = 203 mW.

Figure 3a shows the fluorescence spectrum of the Nd:FAP ceramics in the wavelength range between 800 and 1500 nm. This fluorescence spectrum almost agrees with that of the Nd:FAP single crystal^21^. The sharp emission line occurs at 1063 nm due to the ^4^F_3/2_ to ^4^I_11/2_ electronic transition. The fluorescence decay curve for the emission line is shown in Fig. 3b. From the time-dependence of the fluorescence, the lifetime *τ*_*f*_ determined by a fit of exp(−*t*/*τ*_*f*_), was found to be 0.16 ms, which is close to the value of ~0.2 ms for a single crystal^21^.

For the lasing test, the cavity length was set to be only 1 mm. The details of the laser cavity can be found in the Method section. Figure 3d shows the output power as a function of absorbed pump power *P*_pump_. The pump source was operated at a pulse duration of 1 ms at 10-Hz repetition rate to reduce thermal effects in the laser gain medium. The average output power measured by a thermopile was >10 mW, which corresponds to 1 W peak power, and maximum slope efficiency of 6.5% at *P*_pump_ > 200 mW was obtained. Figure 3e displays an example of the lasing spectrum for single pulse at *P*_pump_ = 203 mW, which shows that the laser wavelength is 1063.5 nm. The relatively narrow linewidth indicates single longitudinal-mode operation.

For the laser characteristics, the laser power increased nonlinearly at *P*_pump_ < 200 mW, as shown in Fig. 3d. In order to understand the behaviour, we examined the average temporal signal of the laser output power using a photo-diode (Supplementary Fig. S2). The integrated temporal intensity (Supplementary Fig. S2) also increased nonlinearly at low pump powers, which agrees with the experimental results obtained separately using the thermopile power meter. One possible reason would be the laser was quasi-CW operation. The laser gain coefficient *g*_0_ is proportional to the number of electrons in the upper laser level which increases exponential term with the ratio of excitation time *t* and lifetime *τ*_*f*_ as 1 − exp(−*t*/*τ*_*f*_)^22^. Since the lifetime of Nd:FAP is *τ*_*f*_ = 0.16 ms, laser gain coefficient *g*_0_ has temporal distribution within excitation time (*t* = 1 ms). This effect may occur to the nonlinearity of average output power and temporal signal shown in Supplementary Fig. S2.

To discuss another possibility, we measured the average lasing spectrum for multi-pulses under various pump conditions (Supplementary Fig. S3). It should be noted that the spectra in Supplementary Fig. S3 are an average of at least 100 pulses, while Fig. 3e displays a single spectral measurement. Since the cavity length is sufficiently short, each pulse must be nearly longitudinally single-mode. It was observed, however, that each oscillation wavelength varies within the 1063 to 1064 nm wavelength-range. A possible reason for this is the fluctuation of the cavity length due to thermal expansion caused by the heat generated during laser oscillation. When the pump power was weak, the possibility of laser oscillation in longer-wavelengths was lowered. Additionally, the laser cavity was composed of flat mirrors and laser cavity was unstable. Therefore, laser oscillation may have been achieved by the thermal lens effect which is dependent on the pump intensity and pump duration.

One may think that laser oscillation with a random crystal orientation will be realized on a similar principle to the random laser effect^23,24^. However, random lasers usually require a material combination with a high refractive index difference to confine light inside the material. Since the refractive index difference of our Nd:FAP is only Δ*n* ~ 0.003, it is unlikely that a confined path occurs in the three-dimensional medium. Although random lasers do not require laser cavity, Nd:FAP ceramics did not achieve laser oscillation when the output coupler was excluded. Furthermore, random lasers should have several longitudinal modes to be identified; our laser operates close to longitudinally single-mode as shown in Fig. 3(e). From the above reasons, we believe that the oscillation of this laser is different from the principle of random laser action.

These aspects are still under investigation, and we are planning to introduce a stable cavity configuration, a sample-cooling system, and an anti-reflective coating to achieve more stable and efficient operation. This will enable the laser characteristics to be investigated in more detail and compared with other laser materials to explore potential applications of Nd:FAP lasers.

## Conclusions

In conclusion, we demonstrated laser oscillation in randomly oriented non-cubic Nd:FAP ceramics. Highly transparent non-cubic ceramics were achieved using both advanced nano-powder synthesis and controlled SPS processes. We produced a highly efficient optical ceramic material with fine grains (140 nm) and high transmittance (87%). Laser oscillation was also demonstrated with a slope efficiency of 6.5% and high output peak power of >1 W.

We plan to improve the optical quality further by optimizing the powder synthesis procedure and sintering conditions; specifically, by separating agglomerated powders by jet milling, improving the degree of vacuum, and adjusting the applied pressure. The laser performance can also be enhanced by depositing an anti-reflective coating on the ceramic surfaces and introducing a cooling system. In addition, introducing other rare-earth active elements (e.g. Er) into the ceramic composition will be examined for industrial and medical mid-infrared laser applications, since a higher laser performance can be achieved when the scattering coefficient decreases at high lasing wavelengths.

Laser ceramics with small grains and random crystal orientation should be also possible for other host materials. We expect that many new non-cubic laser ceramic materials will be realized in the future based on these findings.

## Methods

### Powder synthesis

1 at.% Nd:FAP powder was synthesized by a wet chemical route^25^. From stoichiometric amounts of NdCl_3_·6H_2_O, and analytical grade calcium hydrate dissolved in phosphoric acid, Nd-doped hydroxyapatite (HAP) was first prepared. Then the Nd:HAP powder was mixed with trifluoroacetamide (CF_3_CONH_2_), water and ethanol. The mixture was dried at 60 °C for 24 h and fluorine-substituted by heating at 800 °C for 2 h in air, to synthesize Nd:FAP powder. The synthesized powder was then ground and passed through a 200 mesh sieve to eliminate hard agglomerates. The morphology of the Nd:FAP secondary powder is shown in Supplementary Fig. S4.

### Spark plasma sintering (SPS)

The Nd:FAP powder was consolidated by using an SPS machine (LABOX-315, Sinter Land, Japan) to obtain dense bodies. The powder was poured into a SiC mould with a 10 mm-inner diameter, and uniaxially pressed using SiC punch. A carbon sheet was used between SiC and Nd:FAP powder. The die and punch, which are electrically conductive^13^, were heated at a rate of 5 °C/min under a uniaxial pressure of 80 MPa in vacuum. The sintering temperature was 950 °C and the holding time was 20 min. The temperature was measured using a thermocouple in the non-through hole of the mould. After sintering, both surfaces of the ceramics were mirror-polished. For the observation of microstructure, the polished surfaces were thermally etched at 800 °C for 2 h in air, and coated with Pt.

### Characterization

For the powder and the sintered body, the crystal structures were characterized by X-ray diffraction (XRD; Ultima IV, Rigaku, Japan), and the microstructures were examined using a field-emission scanning electron microscope (FE-SEM; JSM-6701F, JEOL, Japan). The grain size was determined from an average area per grain, counted for >300 grains in the FE-SEM micrographs under an assumption of spherical grains. The grain size was used to estimate the scattering coefficient of the Nd:FAP ceramics using the Rayleigh-Gans-Debye theory as formulated by Apetz and Bruggen^19^.

The optical in-line transmittance spectra *T*(*λ*) of the ceramics was measured using a UV/VIS/NIR spectrometer (UV-3100PC, Shimadzu, Japan). The scattering coefficient *γ*(*λ*) was estimated under an assumption of *γ* = *δ* by using

3 $$T(\lambda )={[1-{R}_{{\rm{av}}}(\lambda )]}^{2}\exp [-\delta (\lambda )t],$$

where *t* is the sample thickness, *R*_av_(*λ*) is the theoretical reflectance obtained from the refractive index dispersion *n*_av_(*λ*) as *R*_av_ = (1 − *n*_av_)^2^/(1 + *n*_av_)^2^. Here *n*_av_ is an average of the refractive indices of ordinary and extraordinary waves, and the dispersion can be obtained using a Sellmeier equation. For calculating the transmitted spectra in the Nd:FAP ceramics using the analysed scattering coefficient, the absorption lines by an electron transition of Nd^3+^ was ignored.

### Spectroscopic properties

The fluorescence spectra were measured using an optical spectrum analyser (Q8383, Advantest, Japan). The sample was pumped by a fibre-coupled laser diode (LD) at a wavelength of 807 nm and pump duration of 5 ms. The lifetime was evaluated from the time-dependent fluorescence decay curve measured using a photo-detector and an oscilloscope. For measuring the decay curve, multiple 850-nm long-pass filters were placed in front of the photo-detector, to remove the scattering of pump light.

### Laser oscillation

The experimental setup for laser oscillation is shown in Fig. 3c. For the lasing test, the cavity length of 1 mm was bound by flat dichroic mirror (DM) and flat output coupler (OC) with a reflectivity of 95%. The sintered Nd:FAP ceramics with a thickness of 1.0 mm without anti-reflection (AR) coating was attached to the DM. A CW 60 W fibre-coupled LD was used as a pump source. The centre wavelength of the LD was 806.8 nm with a spectral width of 2 nm. The core diameter of the fibre was 100 μm, with a numerical aperture (NA) of 0.22. The pump beam was collimated and focused on the Nd:FAP using two coupling lenses of the same focal length. In the experiments, the pump source was driven under a quasi-CW mode with a 1 ms-pulse duration and 10 Hz-repetition rate to suppress any problematic thermal build-up in the Nd:FAP ceramics. The laser output power was measured using a thermopile power meter (3 A, Ophir Optronics, Israel), and the laser spectrum using an optical spectrum analyser (Q8383, Advantest, Japan).

## Supplementary information

Supplementary Information

## Data Availability

The data generated or analysed during the present study are available from the corresponding author upon request.
