# Supplementary material for: Transparent non-cubic laser ceramics with fine microstructure
Source: Sci Rep. 2019 Jul 16;9:10300. doi: 10.1038/s41598-019-46616-8 (PMC6635367; doi:10.1038/s41598-019-46616-8)
Supplement: Supplementary file 1 — Supplementary Information [file 41598_2019_46616_MOESM1_ESM.pdf]

Supplementary Information

Transparent non-cubic laser ceramics with fine microstructure

Hiroaki Furuse*, Naohiro Horiuchi, and Byung-Nam Kim*

I. Supplementary Results

1. Refractive index dispersion of randomly oriented Nd:FAP ceramics

The theoretical transmittance of a material $T(\lambda)$, which is expressed as $(1 - R(\lambda))^2$, can be calculated from the refractive index dispersion $n(\lambda)$ as $R(\lambda) = ((1 - n(\lambda))/(1 + n(\lambda)))^2$. Since apatite has a hexagonal crystal structure and it is optically uniaxial, the refractive index of the optic axis (*c*-axis) is different from that of the other axes, i.e. apatite has two refractive indices n_o and n_e, which are the ordinary and extraordinary refractive indices for the light polarized perpendicular and parallel to the optic axis, respectively. We must therefore consider the average refractive index of n_o and n_e for randomly oriented FAP ceramics.

The refractive index dispersion of single-crystal fluorapatite was measured by Becker et. al using the prism method at 12 discrete wavelengths in the range between 0.436 μm and 2.32 μm[S1]. From the measurement, the coefficients of the Sellmeier equation

$$n^2(\lambda) = D_1 + \frac{D_2}{\lambda^2 - D_3} - D_4\lambda^2 \ ,$$

were obtained as shown in Table 1, where λ (μm) is the wavelength of light. The average refractive index, n_{av} can then be obtained roughly as $(n_o + 2n_e)/3$ (Supplementary Table 1 and Supplementary Fig. S1).

Δn_{max} is the difference in the two refractive indices of the birefringent FAP crystal ($\Delta n_{max} = n_o - n_e$). For an isotropic distribution, the refractive index difference Δn_g used in Eq. (1) in the main text is considered to be equal to the birefringent average for all grain orientations as $\Delta n_g = (2/3)\ \Delta n_{max}$ as discussed in Ref. (S2).

Supplementary Table 1. Sellmeier coefficients of fluorapatite crystal [S1].

	D_1	D_2	D_3	D_4
n_o^2	2.6398	0.0145	0.016	0.0086
n_e^2	2.6297	0.0143	0.016	0.0085
n_{av}^2	2.6331	0.0144	0.016	0.0085
Δn_{max}^2	9.64×10^{-6}	3.45×10^{-7}	0.018	1.48×10^{-7}

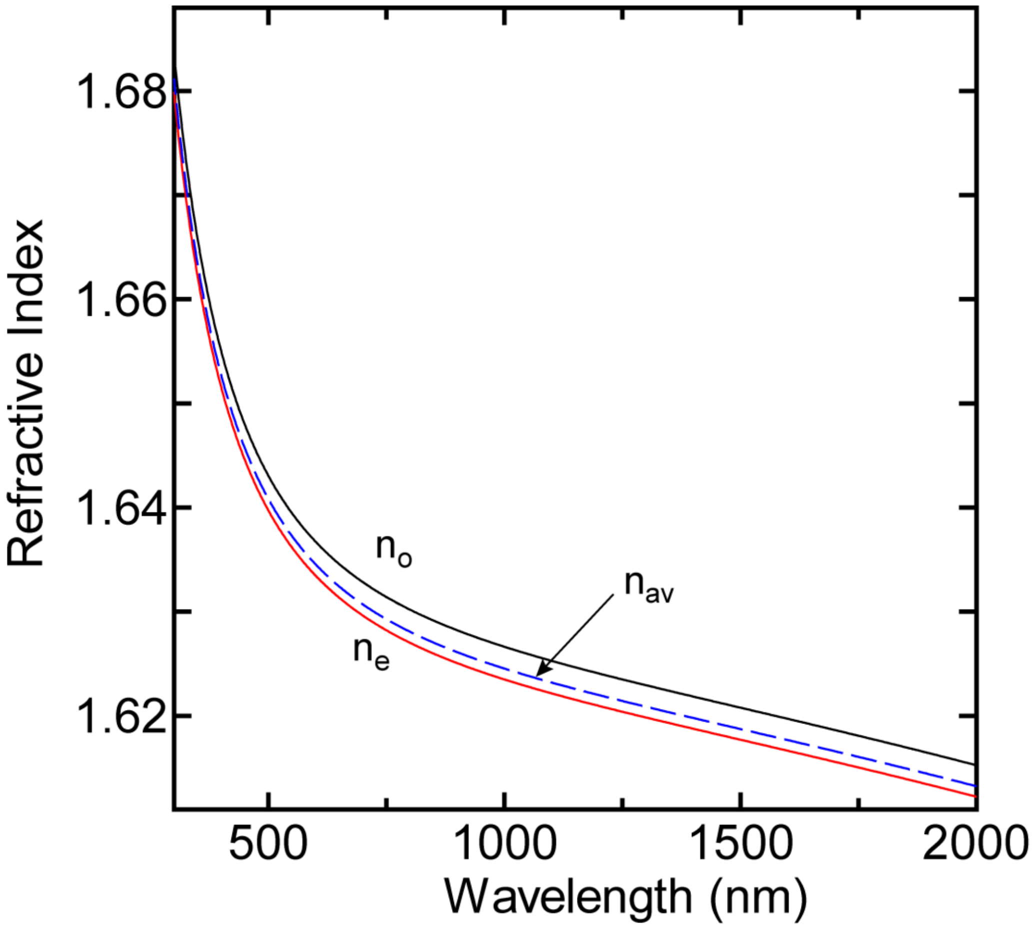

Supplementary Figure 1. Refractive index dispersion of single-crystal fluorapatite reported in Ref. S1. From this data, the average refractive index dispersion $n_{av}(\lambda)$ was obtained by $(n_o + 2n_e)/3$.

2. *Temporal waveform of laser output and lasing spectra for various pump conditions*

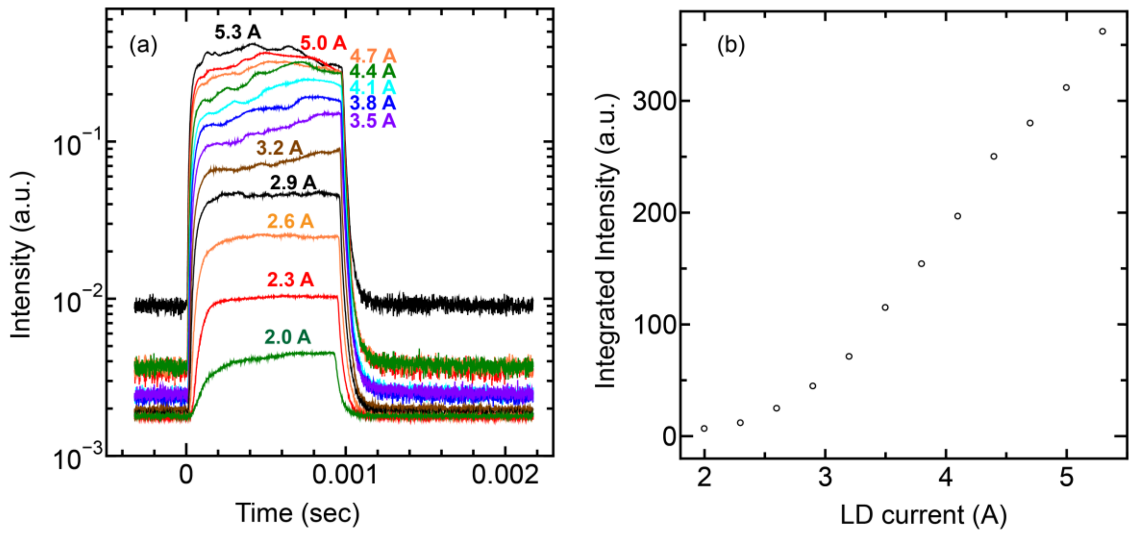

Supplementary Figure 2. **a** Temporal waveform of the laser output, measured using a photo detector and oscilloscope for various pump laser diode (LD) current values. The waveforms are an average of 50 measurements. **b** Temporal waveform intensity values integrated over the time range between 0 and 0.001 s as a function of LD current. The output intensity shows a nonlinearly increasing behaviour.

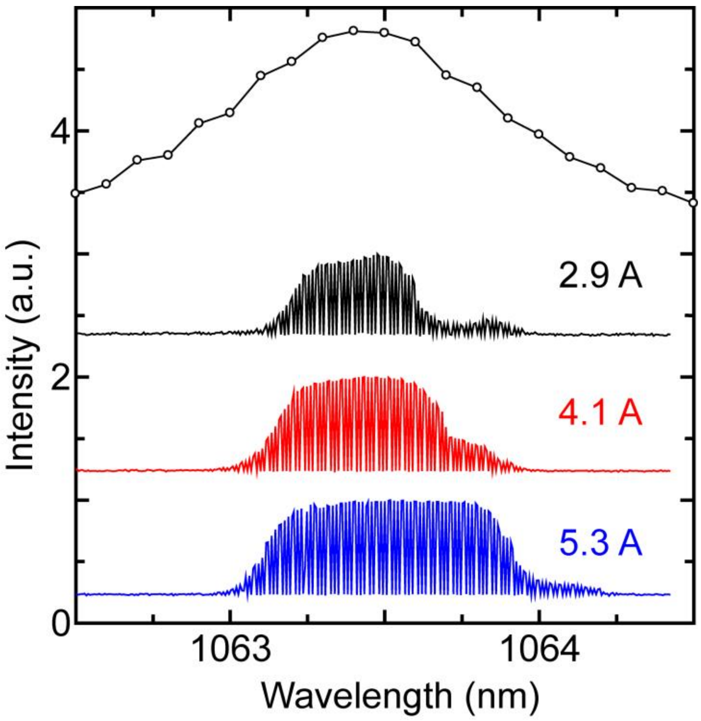

Supplementary Figure 3. Laser oscillation spectra of Nd:FAP ceramics, which is an average of 100 repeated measurements at different LD current conditions. The open-symbol line represents the fluorescence spectral intensity of Nd:FAP as a reference. It is clear that the laser oscillation spectrum fluctuates within the 1063 to 1064 nm wavelength range and the average oscillation intensity > 1063.6 nm is smaller at low pump conditions.

3. *SEM image of the Nd:FAP secondary powder*

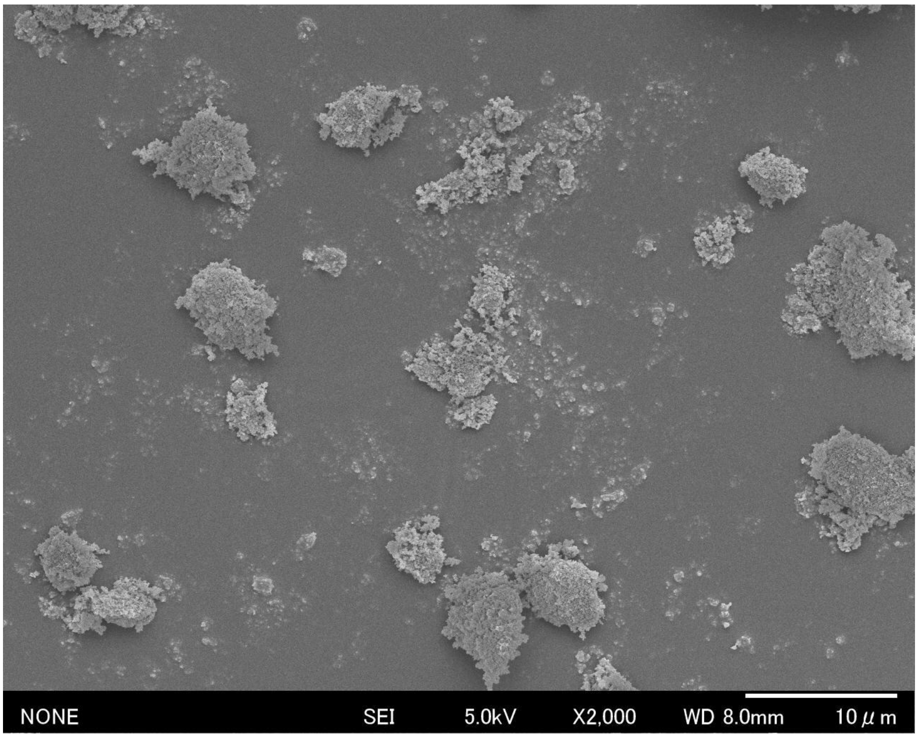

Supplementary Figure 4. SEM image of the Nd:FAP powder after 200 mesh sieving. The size of secondary Nd:FAP powder was several μm.

Supplementary References

[S1] Becker, P., Libowitzky, E., Kleinschrodt, R., & Bohaty, L. Linear optical properties and Raman spectroscopy of natural fluorapatite. *Cryst. Res. Technol.* **51**, 282 (2016).

[S2] Apetz, R., & van Bruggen, M. P. B. Transparent alumina: A light-scattering model. *J. Am. Ceram. Soc.* **86**, 480–486 (2003).
